# Supplementary material for: Interactions with Microbial Proteins Driving the Antibacterial Activity of Flavonoids
Source: Pharmaceutics. 2021 May 5;13(5):660. doi: 10.3390/pharmaceutics13050660 (PMC8147964; doi:10.3390/pharmaceutics13050660)
Supplement: Supplementary file 1 [file pharmaceutics-13-00660-s001.zip › pharmaceutics-1203641-supplementary.pdf]

pharmaceutics

Supplementary Materials: Interactions with Microbial Proteins Driving the Antibacterial Activity of Flavonoids

Giuliana Donadio, Francesca Mensitieri, Valentina Santoro, Valentina Parisi, Maria Laura Bellone, Nunziatina De Tommasi, Viviana Izzo and Fabrizio Dal Pia

Table 1. Antimicrobial flavonoids and their respective molecular targets.

Compound name	Structure	Bacterial strain	Target	Reference
3,6-dihydroxyflavone	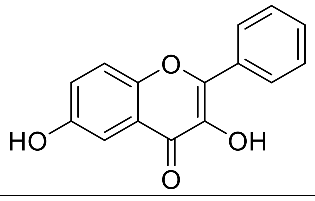	*E. coli*	Inhibition of β-ketoacyl acyl carrier protein (ACP) synthases	[83]
5′-methoxyhydnocarpin	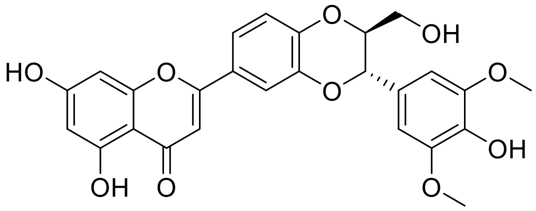	*S. aureus*	Inhibition of MDR-NorA efflux pump	[57]
7,4′-dimethylapigenin	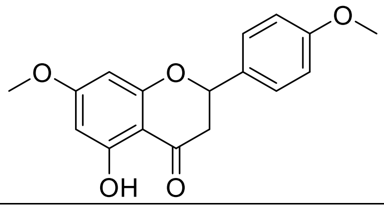	*S. aureus*	Inhibition of NorA efflux pump	[53]
Apigenin	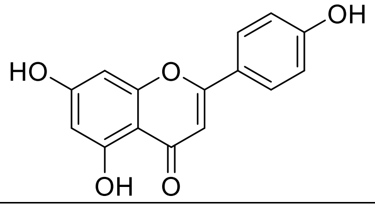	*E. coli*	Partial inhibition of ATP synthase	[81]
		S. aureus	Inhibition of efflux pump	[44]
		S. aureus	Inhibition of NorA efflux pump	[53]
Artonin I	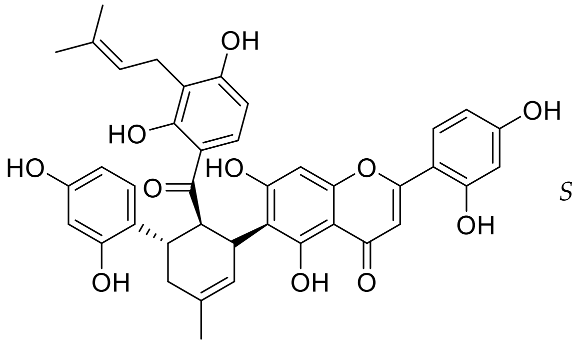	*S aureus*	Inhibition of efflux pump Membrane damage	[56]
Baicalein		*M. smegmatis, M. aurum* and *M. bovis*	Inhibition of efflux pump	[58]
		E. coli	Inhibition of ATP synthase	[81]

	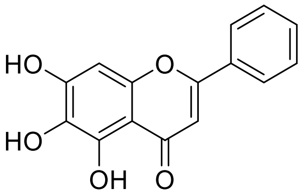	*L. monocytogenes*	Inhibition of listeriolysin O	[98]
		P. aeruginosa	Interaction with LasR and PilT	[109]
Biochanin A	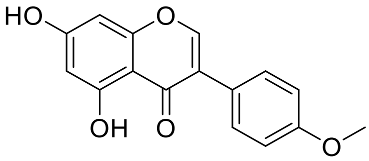	*M. smegmatis*	Inhibition of MDR efflux pump	[44]
		E. coli	Membrane perturbation	[69]
(+)-Catechin	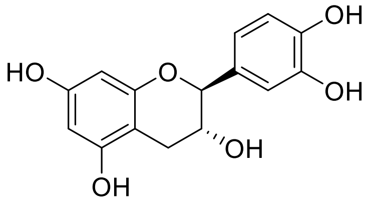	*B. subtilis and E. coli*	ROS regulation Membrane perturbation	[63]
		MRSA MSSA S. aureus	Decrease of SOD and CAT activity	[64]
		E. col	Membrane perturbation	[69]
Chrysin	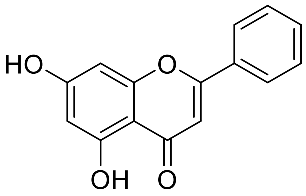	*E. coli*	Partial inhibition of ATP synthase	[81]
		L. monocytogenes	Inhibition of listeriolysin O	[98]
		P. aeruginosa	Interaction with LasR and PilT	[109]
Cirsimaritin	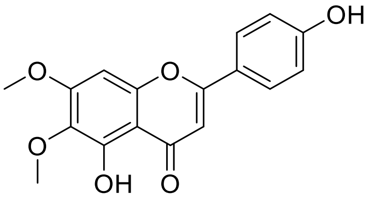	*S. aureus*	Inhibition of NorA efflux pump	[53]
Daidzein	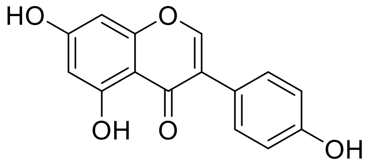	*E. coli*	Weak Inhibition of ATP synthase	[81]
Dihydroquercetin	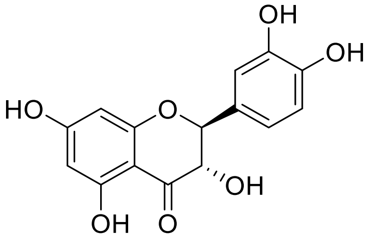	*E. coli and S. aureus*	Inhibition of β-ketoacyl ACP reductase, 3R-hydroxyacyl ACP dehydratase and trans 2- enoyl ACP reductase	[68]
		C. violaceum	Interaction with CviR protein	[112]
Diosmin	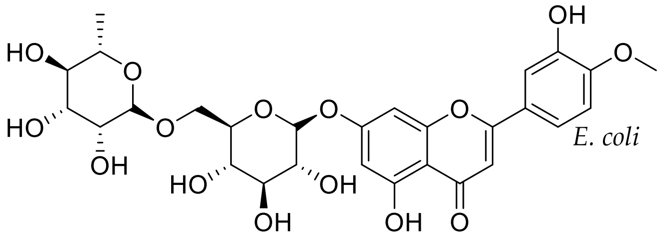	*E. coli*	Partial inhibition of ATP synthase	[81]

(-)-Epicatechin	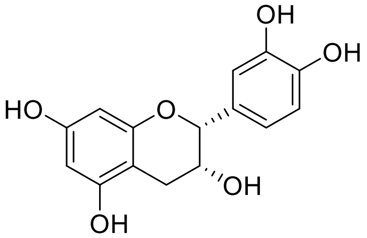	*E. coli*	Inhibition of ATP synthase	[78,81]
(-)-Epicatechin gallate	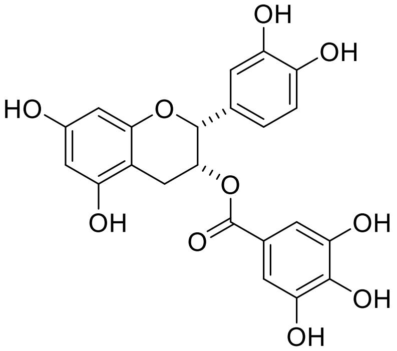	*E. coli*	Inhibition of DNA gyrase (sub GyrB)	[115]
(-)-Epigallocatechin	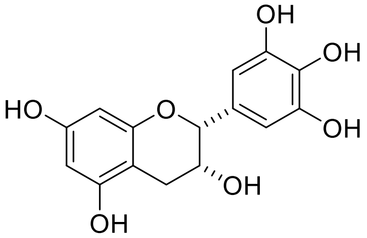	*E. coli*	Inhibition of DNA gyrase (sub GyrB)	[115]
(-)-Epigallocatechin gallate	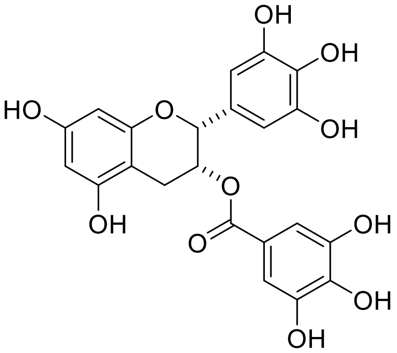	*E. coli*	Inhibition of DNA gyrase (sub GyrB)	[115]
Fisetin	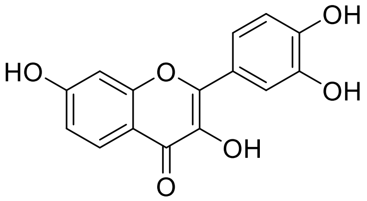	*L. monocytogenes*	Inhibition of listeriolysin O	[98]
Flavone	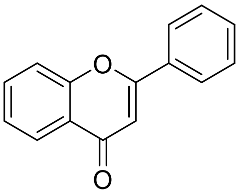	*S. aureus*	Inhibition of expression of the gene encoding for α-hemolysin (Hla) Inhibition of staphyloxanthin production	[96]
Galangin	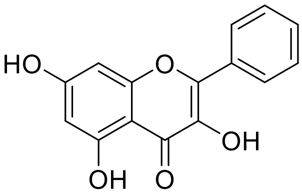	*E. coli*	Weak inhibition of ATP synthase	[81]

Genistein	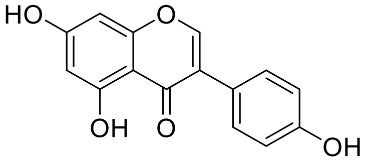	*E. coli*	Partial Inhibition of ATP synthase	[81]
Genkwanin	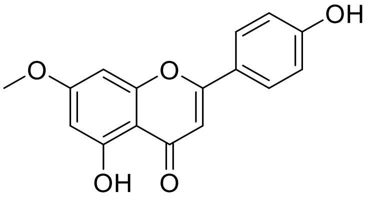	*S. aureus*	Inhibition of NorA efflux pump	[53]
Hesperidin	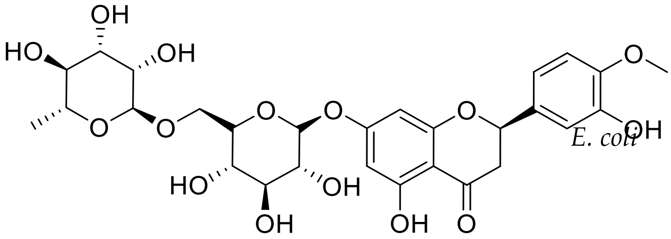	*E. coli*	Partial inhibition of ATP synthase	[81]
Hesperetin	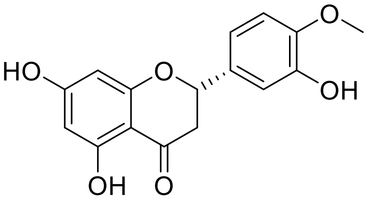	*E. coli*	Membrane perturbation	[69]
Kaempferol	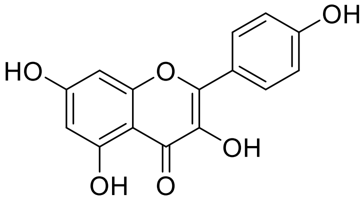	*E. coli*	Partial inhibition of ATP synthase	[81]
		E. coli and S. aureus	Membrane perturbation	[68]
		E. coli		[69]
		S. aureus	Inhibition of efflux pump	[52]
Kurarinol	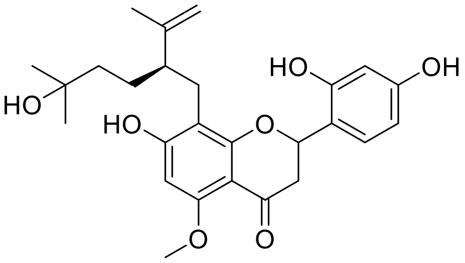	*S aureus*	Inhibition of Sortase A	[91]
Leucodelphinidin	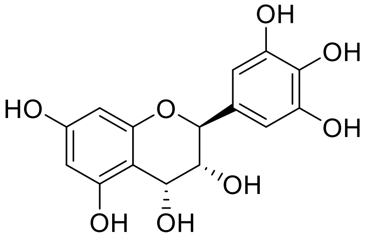	*E. coli and S. aureus*	Inhibition of β-ketoacyl ACP reductase, 3R-hydroxyacyl ACP dehydratase and trans 2- enoyl ACP reductase	[68]

Lonicerin	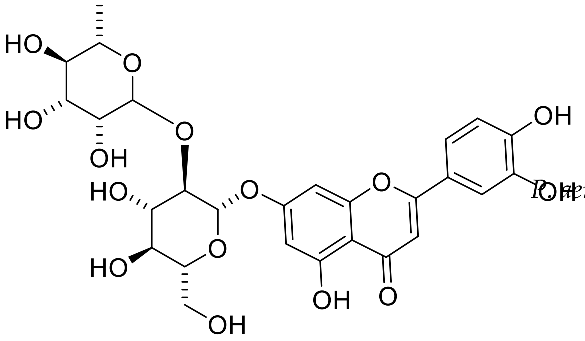	*P. aeruginosa*	Inhibition of alginate production protein AlgE	[92]
Luteolin	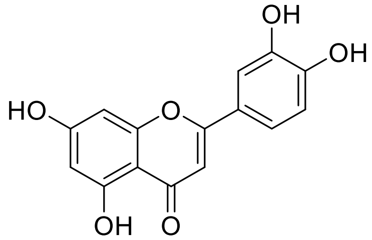	*E. coli*	Weak inhibition of ATP synthase	[81]
		S. aureus	Inhibition of staphyloxanthin production	[96]
		E. coli	Inhibition of assembly of amyloid curli fibers	[111]
Morin	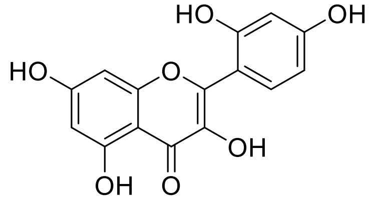	*E. coli*	Inhibition of ATP synthase	[78,79,81]
		L. monocytogenes	Inhibition of listeriolysin O	[98]
		E. coli	Inhibition of assembly of amyloid curli fibers	[111]
Morusin	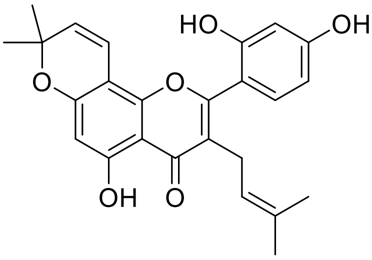	*S. aureus* and *S. enterica*	Downregulation of expression levels of different subunit of the fatty-acid-synthase (fabD, fabF, fabG and fabH)	[74]
Myricetin	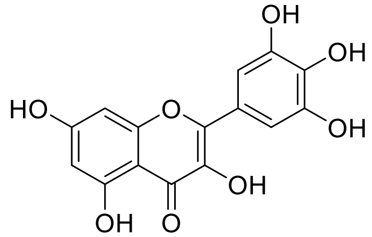	*L. monocytogenes*	Inhibition of listeriolysin O	[98]
		S. aureus	Inhibition of efflux pump	[44,51]
		E. coli	Inhibition of assembly of amyloid curli fibers	[111]
		E. coli	Inhibition of cellular functions of DnaK	[102]
Naringenin	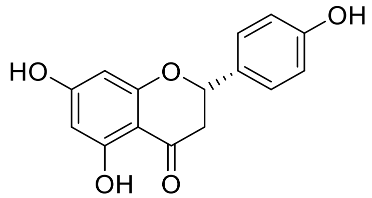	*B. subtilis*	Inhibition of assembly of amyloid curli fibers	[111]
Nobiletin	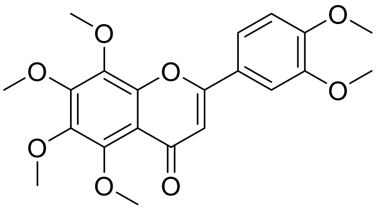	*M. smegmatis*, *M. aurum*, and *M. bovis*	Inhibition of efflux pump	[58]
Phloretin	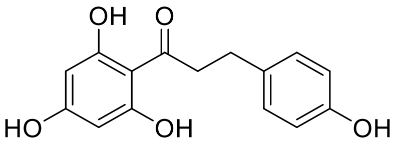	*B. subtilis*	Inhibition of assembly of amyloid curli fibers	[111]

Quercetin	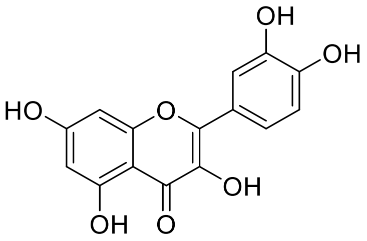	*E. coli and S. aureus*	Inhibition of β-ketoacyl ACP reductase, 3R-hydroxyacyl ACP dehydratase and trans 2- enoyl ACP reductase	[68]
		E. coli	Inhibition of assembly of amyloid curli fibers	[111]
		E. coli	Inhibition DNA gyrase (sub GyrB)	[72]
		E. coli	Inhibition of ATP synthase	[78,79]
		P. aeruginosa	Interaction with PilT and LasR	[109]
Rhamnetin	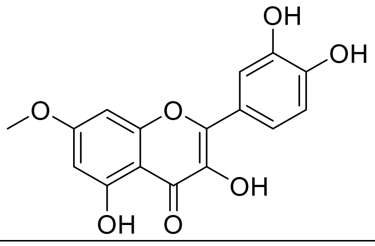	*S. aureus*	Inhibition of efflux pump	[52]
Rutin	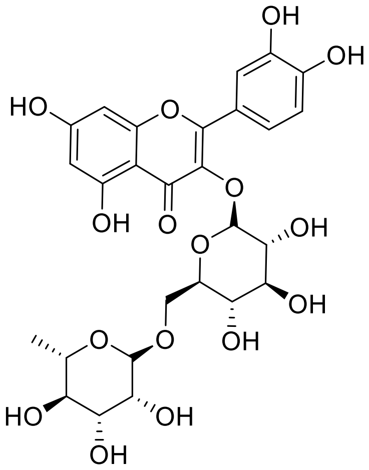	*E. coli*	Partial inhibition of ATP synthase	[81]
Silibinin	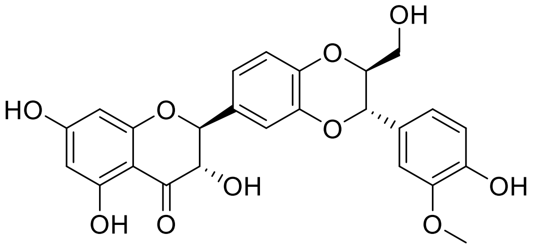	*E. coli*	Inhibition of ATP synthase	[81]
Silymarin	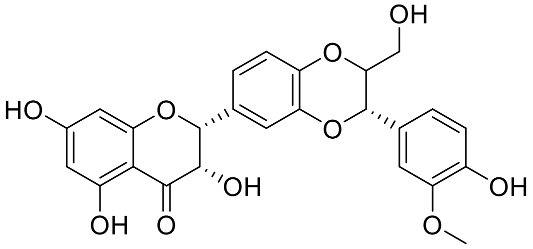	*E. coli*	Inhibition of ATP synthase	[81]
Skullcapflavone II	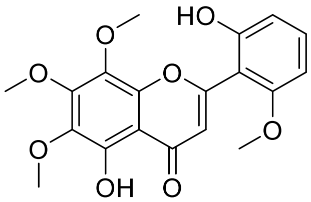	*M. smegmatis, M. aurum*, and *M. bovis*	Inhibition of efflux pump	[58]

Tangeretin	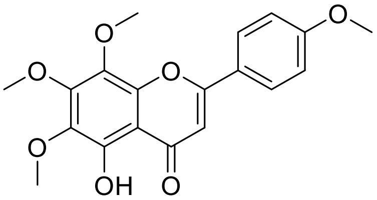	*M. smegmatis*, *M. aurum*, and *M. bovis*	Inhibition of efflux pump	[58]
Tetramethylscutellarein	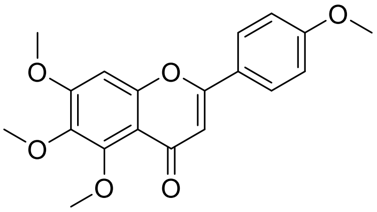	*S. aureus*	Inhibition of NorA efflux pump	[53]
Trimethylapigenin	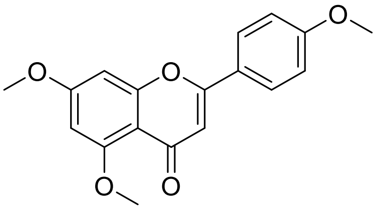	*S. aureus*	Inhibition of NorA efflux pump	[53]
Wogonin	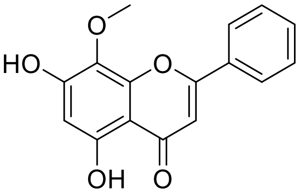	*M. smegmatis*, *M. aurum*, and *M. bovis*	Inhibition of efflux pump	[58]
